# Pulsed Radiofrequency Alone Versus Combined With Stellate Ganglion Block for Acute Herpes Zoster–Associated Trigeminal Neuralgia in Patients With Pain Catastrophizing: A Retrospective Cohort Analysis

**DOI:** 10.1155/prm/4344647

**Published:** 2026-09-16

**Authors:** Chengwen Wu, Qi Zhou, Yue Zhang, Cehua Ou

**Affiliations:** ^1^ Department of Pain Management, The Affiliated Hospital of Southwest Medical University, Luzhou, China, ahswmu.cn; ^2^ Department of Obstetrics, Luzhou Maternal and Child Health Hospital, Luzhou, China

**Keywords:** herpes zoster–associated trigeminal neuralgia, pain catastrophizing, Pittsburgh sleep quality index, pulsed radiofrequency, stellate ganglion block, visual analog scale

## Abstract

**Purpose:**

This study compared the efficacy of pulsed radiofrequency (PRF) combined with stellate ganglion block (SGB) versus PRF alone in managing acute herpes zoster–associated trigeminal neuralgia (HZTN) with pain catastrophizing.

**Patients and Methods:**

Between 2019 and 2024, 61 patients were divided into two groups: the PRF group (supraorbital nerve PRF, *n* = 31) and the PRF + SGB group (PRF combined with SGB, *n* = 30). Pain (VAS), sleep quality (PSQI), and pain catastrophizing (PCS) were evaluated at baseline, at 1 and 2 weeks, and at 1, 3, and 6 months post‐treatment. Adverse events and satisfaction were also tracked.

**Results:**

The PRF + SGB group showed better pain relief and sleep improvement within the first 3 months (*p* < 0.05) but not at 6 months. Both groups improved in PCS scores (*p* < 0.05), but PRF + SGB had consistently lower scores at all follow‐ups (*p* < 0.05). Multivariable regression showed that the treatment group was independently associated with 6‐month PCS, but not with 6‐month VAS or PSQI. Patient satisfaction was higher in the PRF + SGB group (*p* < 0.05). No significant adverse events occurred in either group.

**Conclusion:**

Combining PRF with SGB was associated with greater short‐term efficacy in reducing pain, improving sleep, and relieving pain catastrophizing, with higher patient satisfaction and a favorable safety profile. However, the benefits in pain and sleep were not sustained at 6 months, suggesting mainly early clinical benefit and a persistent association with improved pain catastrophizing.

## 1. Introduction

Herpes zoster–associated trigeminal neuralgia (HZTN) is an acute neuropathic pain syndrome triggered by the reactivation of the varicella‐zoster virus, characterized by severe, lancinating, or burning pain that often affects the ophthalmic division of the trigeminal nerve [1]. HZTN not only represents a significant acute pain condition but is also frequently associated with substantial psychological distress and sleep disturbances, particularly in patients exhibiting pain catastrophizing [2]. Pain catastrophizing refers to the negative cognitive processing of actual or anticipated pain experiences, which not only intensifies pain perception but also undermines coping abilities [3, 4]. Consequently, identifying a treatment approach that effectively alleviates pain while simultaneously improving psychological well‐being is crucial for patients suffering from this condition.

Pulsed radiofrequency (PRF) is a minimally invasive therapeutic modality widely utilized in the management of neuropathic pain. It involves delivering intermittent electrical currents to target nerves, thereby inhibiting abnormal neuronal discharges and reducing pain symptoms. PRF has gained prominence due to its ability to modulate neural function without causing permanent nerve damage, making it a preferred option for managing trigeminal neuralgia secondary to herpes zoster [5–9]. However, although PRF demonstrates efficacy in many cases, it may not be sufficient as a standalone treatment to fully alleviate symptoms in patients with pain catastrophizing, where psychological factors play a significant role in shaping the pain experience. Stellate ganglion block (SGB), another commonly employed intervention for pain management, involves administering local anesthetics to the stellate ganglion within the cervical sympathetic chain. This procedure improves blood circulation, reduces sympathetic hyperactivity, and provides relief for head and facial pain, as well as upper limb discomfort [10–12]. Recent studies have highlighted the potential of SGB beyond pain control, demonstrating its efficacy in psychiatric conditions, such as post‐traumatic stress disorder (PTSD) [11, 13]. These findings suggest that SGB may not only mitigate pain but also address the psychological aspects associated with chronic pain syndromes, including pain catastrophizing.

In our previous study [14], we compared the efficacy of peripheral nerve stimulation (PNS) with PRF and found that the PNS group showed superior improvement in pain catastrophizing across all follow‐up time points. Given the dual therapeutic effects of SGB on psychological and pain‐related outcomes, a retrospective analysis was conducted to compare the efficacy of the PRF + SGB combination versus PRF monotherapy. This investigation aimed to determine whether SGB could mitigate the limitations of PRF in reducing pain catastrophizing, thereby offering a cost‐effective and versatile treatment strategy. Furthermore, we evaluated the impact of these two interventions on pain intensity, sleep quality, and patient satisfaction during short‐ and long‐term follow‐up periods. A systematic analysis of adverse events following treatment was also performed to comprehensively assess the safety and effectiveness of these approaches. Through this integrated evaluation, we aim to provide robust scientific evidence to guide clinical decision‐making, optimize therapeutic outcomes, and ultimately enhance the quality of life for patients with acute HZTN and pain catastrophizing.

## 2. Material and Methods

### 2.1. Participants

**FIGURE 1:**
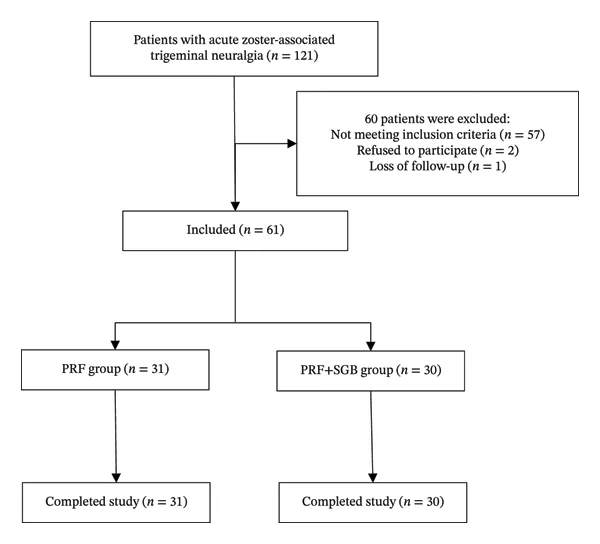
Workflow diagram of this study. Abbreviations: PRF: pulsed radiofrequency; SGB: stellate ganglion block.

This retrospective analysis was conducted at the Department of Pain Management, Affiliated Hospital of Southwest Medical University, with data collection spanning from March 2019 to June 2024. Inclusion criteria were as follows: (1) acute HZTN involving the first branch of the trigeminal nerve with disease duration ≤ 1 month; (2) failure to achieve significant pain relief after conservative treatments including pregabalin and transcutaneous electrical nerve stimulation (TENS); (3) Pain Catastrophizing Scale (PCS) score ≥ 30; and (4) visual analog scale (VAS) score ≥ 5. Exclusion criteria included: (1) local skin ulcers or infections; (2) severe cardiopulmonary, hepatic, or renal dysfunction; (3) psychiatric disorders; (4) coagulation disorders or anticoagulant therapy; (5) history of drug abuse; (6) psychological therapy; and (7) other invasive treatments, such as peripheral nerve electrical stimulation, trigeminal ganglion electrical stimulation, or trigeminal ganglion radiofrequency thermocoagulation. Of the 121 screened patients, 61 met the inclusion and exclusion criteria and were analyzed. They were divided into two groups based on treatment modality: the PRF group (*n* = 31) and the PRF + SGB group (*n* = 30) (Figure 1). All medical records were thoroughly reviewed to extract clinical data before and after treatment. Ethical approval was obtained from the Clinical Trial Ethics Committee of the Affiliated Hospital of Southwest Medical University (approval number: KY2025071). The requirement for written informed consent was waived by the ethics committee due to the retrospective nature of the study and the use of anonymized medical records, which posed no risk to patient privacy. This study was conducted in accordance with the Declaration of Helsinki.

### 2.2. Description of Procedures

#### 2.2.1. Ultrasound‐Guided Supraorbital Nerve PRF

**FIGURE 2:**
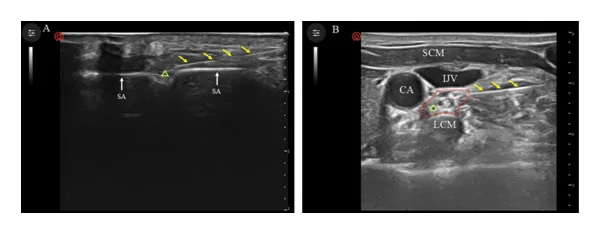
Diagram illustrating the procedure for supraorbital nerve PRF and SGB. (A) The yellow triangle indicates the supraorbital notch; the white arrow points to the supraorbital arch; the yellow arrow indicates the needle for puncture. (B) The yellow triangle marks the area of the stellate ganglion; the yellow arrow indicates the puncture needle; the red dotted line within the orbit represents the distribution area of the injected medication. Abbreviations: SA: supraorbital arch, LCM: longus colli muscle, IJV: internal jugular vein, CA: common carotid artery, SCM: sternocleidomastoid.

Patients were placed in a supine position with a soft pillow under their heads. Using ultrasound (Terason t3000 TM Ultrasound System, Teratech Corporation, Burlington, MA, USA), the target area was located by observing the continuous hyperechoic shadow of the supraorbital ridge (Figure 2A). The interruption of this hyperechoic shadow indicated the location of the supraorbital foramen or notch. After marking the puncture site, the area was sterilized, and the ultrasound probe was covered with a sterile sheath. Local infiltration anesthesia was administered using 1% lidocaine. Under ultrasound guidance, a radiofrequency electrode needle (model 20G, Innoman Medical Technology Co., Ltd.) was inserted into the target area. Electrical stimulation (0.2–0.3 V, 50 Hz) was applied to induce numbness in the forehead region, confirming needle placement. Subsequently, PRF treatment was performed with the following parameters: 70 V, 2 Hz, 20 ms, 40°C, 6 min; and 70 V, 2 Hz, 20 ms, 42°C, 6 min. Both groups underwent three sessions of radiofrequency treatment during hospitalization, spaced 1 day apart.

#### 2.2.2. Ultrasound‐Guided SGB

Patients were positioned supine with a thin pillow under the neck, turning their head to the opposite side to expose the neck fully. A high‐frequency probe was used to scan the C6/C7 transverse processes, identifying the common carotid artery, internal jugular vein, and longus colli muscle. The slightly thickened moderate‐to‐strong echo area lateral to the carotid artery and medial to the longus colli muscle was identified as the stellate ganglion (Figure 2B). After sterilization and draping, an in‐plane needle insertion technique was used, carefully avoiding nearby vessels. After penetrating the prevertebral fascia, 5 mL of 1% lidocaine was injected slowly to ensure proper diffusion between the longus colli muscle and the carotid artery. The presence of Horner’s syndrome (e.g., miosis and ptosis) or complications, such as hoarseness and upper limb numbness, was monitored. The PRF + SGB group received SGB treatment daily for 5 consecutive days during the three radiofrequency sessions.

#### 2.2.3. Follow‐Up and Outcome Measures

Follow‐up assessments were conducted by independent researchers blinded to the group assignments and not involved in treatment. Assessments were performed at baseline and at 1 week, 2 weeks, 1 month, 3 months, and 6 months post‐treatment. The following measures were evaluated.

### 2.3. VAS

The intensity of pain was measured using a 10 cm line where one end represented “no pain” and the other “worst possible pain.” Scores were converted to a range of 0 (no pain) to 10 (worst pain).

### 2.4. PCS

This 13‐item questionnaire measures catastrophic thinking, with each item scored from 0 to 4. Total scores range from 0 to 52, with scores ≥ 30 indicating high levels of catastrophic thinking. PCS scores were recorded at baseline and at 1 month, 3 months, and 6 months post‐treatment.

### 2.5. Pittsburgh Sleep Quality Index (PSQI)

This 19‐item self‐rated and 5‐item other‐rated questionnaire evaluates sleep quality. Only the first 18 items contribute to scoring, divided into seven components: subjective sleep quality, sleep latency, sleep duration, sleep efficiency, sleep disturbances, use of sleeping medications, and daytime dysfunction. Each component is scored from 0 to 3, yielding a total score ranging from 0 to 21. Higher scores indicate poorer sleep quality. PSQI scores were recorded at baseline and at 1 week, 2 weeks, 1 month, 3 months, and 6 months post‐treatment.

### 2.6. Patient Satisfaction

Patient satisfaction was assessed at 6 months post‐treatment and categorized as follows: 1, dissatisfied; 2, neutral; and 3, satisfied.

### 2.7. Adverse Events

Intraoperative and postoperative adverse events, including temporary (e.g., infection, hematoma, pneumothorax) and permanent (e.g., corneal injury, recurrent laryngeal nerve damage) complications, were recorded.

### 2.8. Statistical Analysis

Statistical analyses were performed using SPSS 27.0 (IBM Corp., Armonk, NY, USA) and Prism 10.1.2 (GraphPad, San Diego, CA, USA). Normally distributed continuous data are presented as mean ± standard deviation (SD); non‐normal data are described as median (interquartile range, IQR). Categorical data are reported as frequencies and percentages. Before analysis, statistical assumptions were rigorously assessed. Longitudinal changes were assessed using two‐way repeated‐measures ANOVA with Sidak’s post hoc tests. If sphericity was violated (Mauchly’s *p* < 0.05), results were corrected using Greenhouse–Geisser epsilon‐adjusted *p* values. For non‐normal data in repeated‐measures designs, we applied a Box–Cox transformation; if normality was achieved, ANOVA was performed. If transformation failed, the Friedman test was used as a nonparametric alternative. Count data were analyzed using the chi‐square test, and ranked data were compared using the rank‐sum test. Multivariable linear regression analysis was performed to assess the independent effects of multiple predictors on the outcome variable, adjusting for potential confounding factors. *p* < 0.05 was considered statistically significant.

## 3. Results

### 3.1. Baseline Characteristics of Participants

A total of 61 patients completed the study successfully. Baseline characteristics are summarized in Table 1. No statistically significant differences were observed between the two groups in terms of age, gender, disease duration, pain severity, pain catastrophizing, or sleep quality, ensuring similar preoperative features across groups.

**TABLE 1:** The baseline characteristics of the patients.

Baseline characteristics	PRF group (*n* = 31)	PRF + SGB group (*n* = 30)	*p* value
Age (years)	60.10 ± 16.38	65.93 ± 15.39	0.145
Gender (male/female)	15/16	18/12	0.363
Pain duration (days)	17.26 ± 9.02	19.63 ± 9.32	0.329
VAS score	7.19 ± 0.98	7.30 ± 0.99	0.598
PCS score	37.62 ± 2.89	37.51 ± 3.02	0.994
PSQI score	13.65 ± 2.25	13.70 ± 2.66	0.948

### 3.2. Comparison of VAS Scores at Different Postoperative Time Points

**FIGURE 3:**
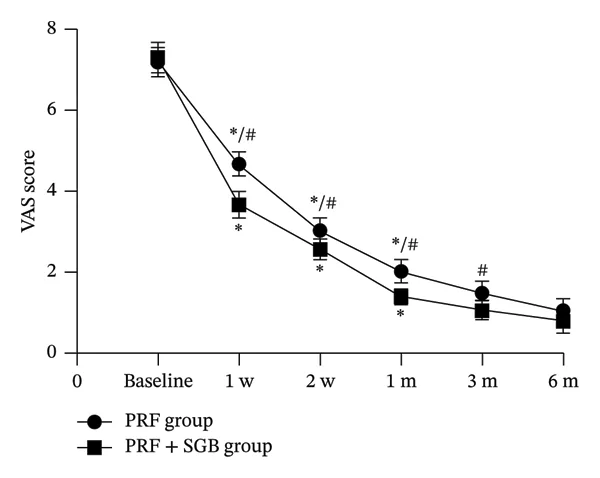
Comparison of VAS scores. ^*#*^ *p* < 0.05, compared with the PRF + SGB group. ^∗^ *p* < 0.05, compared with the preceding time point within the same group.

Both groups showed significant reductions in VAS scores post‐treatment (*p* < 0.05; Figure 3). At baseline, there were no significant differences in VAS scores between the two groups (PRF group: 7.19 ± 0.980 vs. PRF + SGB group: 7.30 ± 0.988, *p* = 0.598). In the early postoperative phase (1 week, 2 weeks, 1 month, and 3 months), the PRF + SGB group exhibited significantly greater reductions in VAS scores compared with the PRF group (1 week: 4.68 ± 0.626 vs. 3.67 ± 0.782, *p* < 0.001; 2 weeks: 3.03 ± 0.699 vs. 2.57 ± 0.679, *p* = 0.041; 1 month: 2.03 ± 0.795 vs. 1.40 ± 0.498, *p* = 0.001; 3 months: 1.48 ± 0.811 vs. 1.07 ± 0.640, *p* = 0.018). However, at the 6‐month follow‐up, no significant intergroup differences were observed (PRF group: 1.06 ± 0.814 vs. PRF + SGB group: 0.80 ± 0.805, *p* = 0.140).

### 3.3. Changes in Pain Catastrophizing Thoughts

**FIGURE 4:**
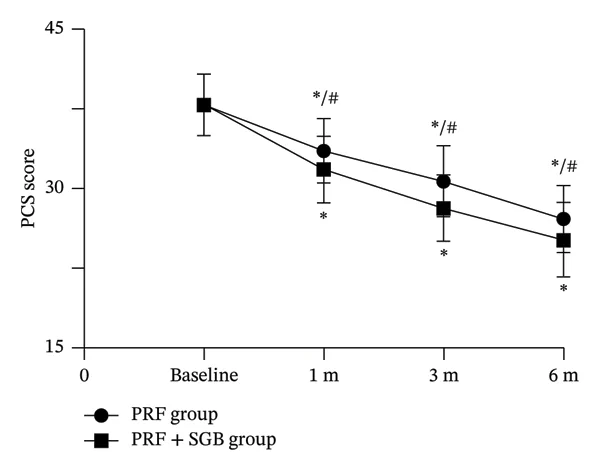
Comparison of PCS scores. ^*#*^ *p* < 0.05, compared with the PRF + SGB group. ^∗^ *p* < 0.05, compared with the preceding time point within the same group.

Baseline PCS scores were similar between groups (PRF group: 37.62 ± 2.89 vs. PRF + SGB group: 37.51 ± 3.02, *p* = 0.994). Post‐treatment, both groups showed significant reductions in PCS scores at all time points (*p* < 0.05; Figure 4). The PRF + SGB group consistently showed significantly lower PCS scores than the PRF group at 1 month (33.52 ± 3.043 vs. 31.73 ± 3.129, *p* = 0.028) and 3 months (30.65 ± 3.332 vs. 28.10 ± 3.133, *p* = 0.003). At the 6‐month follow‐up, the difference remained statistically significant (27.10 ± 3.177 vs. 25.13 ± 3.511, *p* = 0.025).

### 3.4. Sleep Quality Assessment

**FIGURE 5:**
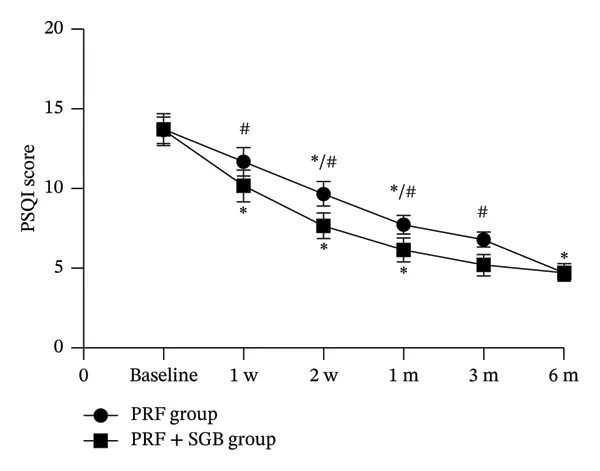
Comparison of PSQI scores. ^*#*^ *p* < 0.05, compared with the PRF + SGB group. ^∗^ *p* < 0.05, compared with the preceding time point within the same group.

Baseline PSQI scores were comparable between the PRF group (13.65 ± 2.259) and PRF + SGB group (13.70 ± 2.667; *p* = 0.948). At 1 week post‐treatment, the PRF + SGB group showed a significant improvement in PSQI scores compared with baseline (10.17 ± 2.666 vs. 13.70 ± 2.667, *p* = 0.022), whereas the PRF group showed no significant change (11.68 ± 2.427 vs. 13.65 ± 2.259, *p* = 0.092). By 2 weeks and 1 month post‐treatment, both groups exhibited significant PSQI improvements compared with baseline (2 weeks: PRF group 9.68 ± 2.088 vs. 13.65 ± 2.259, *p* < 0.001; PRF + SGB group 7.67 ± 2.155 vs. 13.70 ± 2.667, *p* < 0.001; 1 month: PRF group 7.74 ± 1.570 vs. 13.65 ± 2.259, *p* < 0.001; PRF + SGB group 6.17 ± 2.019 vs. 13.70 ± 2.667, *p* < 0.001). Notably, the PRF + SGB group consistently achieved lower PSQI scores than the PRF group at all early follow‐ups (2 weeks: 9.68 ± 2.088 vs. 7.67 ± 2.155, *p* < 0.001; 1 month: 7.74 ± 1.570 vs. 6.17 ± 2.019, *p* < 0.001). At 3 months, the PRF + SGB group maintained significantly better sleep quality (6.81 ± 1.276 vs. 5.20 ± 1.769, *p* < 0.001). However, at the 6‐month follow‐up, no significant intergroup differences were observed ([4.00 (4.00, 5.00)] vs. [4.00 (4.00, 5.00)], *p* = 0.952; Figure 5).

### 3.5. Multivariable Regression Analysis of 6‐Month Clinical Outcomes

**FIGURE 6:**
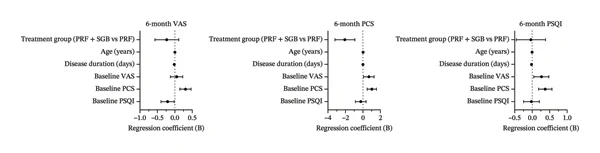
Adjusted forest plot of multivariable linear regression models for 6‐month VAS, PCS, and PSQI outcomes. Points represent adjusted unstandardized regression coefficients (B), and horizontal bars represent approximate 95% confidence intervals.

Multivariable linear regression analyses were conducted to evaluate the associations between the treatment group and 6‐month VAS, PCS, and PSQI after adjustment for age, disease duration, baseline VAS, baseline PCS, and baseline PSQI. For 6‐month VAS, the overall model was significant (*R* ^2^ = 0.396, adjusted *R* ^2^ = 0.329, *p* < 0.001), but the treatment group was not independently associated with the outcome (*B* = −0.231, *p* = 0.192; Fig. 6). Baseline PCS (*B* = 0.307, *p* < 0.001) and baseline PSQI (*B* = −0.207, *p* = 0.033) were significant predictors. For 6‐month PCS, the model showed good explanatory ability (R^2^ = 0.636, adjusted *R* ^2^ = 0.595, *p* < 0.001). The treatment group remained significantly associated with 6‐month PCS (*B* = −2.091, *p* < 0.001), indicating lower PCS scores in the PRF + SGB group compared with the PRF group after adjustment. Baseline VAS (*B* = 0.680, *p* = 0.027) and baseline PCS (*B* = 1.026, *p* < 0.001) were also significant predictors. For 6‐month PSQI, the model was significant (*R* ^2^ = 0.687, adjusted *R* ^2^ = 0.652, *p* < 0.001), but the treatment group was not significantly associated with the outcome (*B* = −0.033, *p* = 0.878). Baseline VAS (*B* = 0.267, *p* = 0.018) and baseline PCS (*B* = 0.377, *p* < 0.001) were significant positive predictors. Overall, after adjustment for potential confounders, PRF + SGB was independently associated with lower 6‐month PCS scores, whereas no significant treatment effect was observed for 6‐month VAS or PSQI.

### 3.6. Patient Satisfaction

At the 6‐month follow‐up, 8 patients (25.8%) in the PRF group and 17 patients (56.7%) in the PRF + SGB group reported satisfaction with treatment outcomes. The PRF + SGB group reported significantly higher satisfaction than the PRF group (8/31, 25.8% vs. 17/30, 56.7%, *χ* ^2^ = 6.201, *p* = 0.045; Table 2).

**TABLE 2:** Patient satisfaction levels between the two groups.

Group	Cases (*n*)	Patient’s satisfaction		
		Dissatisfied (*n*, %)	Neutral (*n*, %)	Satisfied (*n*, %)
PRF group	31	5 (16.1%)	18 (58.1%)	8 (25.8%)
PRF + SGB group	30	2 (6.7%)	11 (36.7%)	17 (56.7%)
*p* value				0.045

### 3.7. Adverse Events

No cases of infection, bleeding, nerve damage, or other adverse effects were observed in either group. No patients discontinued treatment due to adverse reactions.

## 4. Discussion

This study comparatively evaluated the comprehensive therapeutic efficacy of PRF combined with SGB versus PRF alone in acute HZTN patients with pain catastrophizing. The selection of the supraorbital nerve as the PRF target is grounded in robust anatomical and pathophysiological rationale. As the primary branch of the ophthalmic division of the trigeminal nerve, the supraorbital nerve directly innervates the forehead and upper eyelid regions, with its superficial anatomical position facilitating precise ultrasound‐guided localization [5, 9, 15]. PRF modulates abnormal electrical signal transmission through high‐frequency pulsed currents‐generated electric fields, effectively blocking nociceptive signal propagation. It concurrently reduces local inflammatory mediators (e.g., IL‐6 and TNF‐α) and upregulates neural repair‐associated proteins (e.g., ATF3), thereby promoting neural recovery [16, 17]. Compared with PRF targeting the trigeminal ganglion, this peripheral branch intervention preserves proprioceptive function while avoiding deep neural structure damage, significantly reducing puncture‐related complication risks—a critical consideration for maintaining facial sensory integrity. The rationale for combining SGB stems from its dual regulatory effects on neuropathic pain and psychological dimensions. During acute phases, SGB alleviates sympathetic hyperactivity by blocking cervicothoracic sympathetic ganglia, improving local tissue hypoxia and microcirculation, while reducing inflammatory mediator‐induced pain through vasospasm relief [12, 18]. Crucially, SGB directly suppresses sympathetic hyperactivity and reduces the release of norepinephrine (NE), a catecholamine‐like neurotransmitter involved in the activation of pain‐sensitive nerves, regulation of body temperature and sweating, and the activation of the amygdala (the brain’s fear‐processing center). The reduction in NE levels may alleviate anxiety and stress responses [19]. Studies have shown that in patients with PTSD, SGB significantly attenuates hyperactivation of the amygdala, which may be attributed to SGB’s ability to lower NE levels and indirectly dampen amygdala overactivation [11]. The laterocapsular division of the central nucleus (CeLC) and basolateral amygdala (BLA) are critical regions for processing the emotional dimension of pain. In chronic pain models, enhanced excitatory synaptic transmission in the CeLC, coupled with impaired inhibitory interneuron (ITC) function, leads to an imbalanced excitation‐inhibition (E/I) ratio, contributing to anxiety and pain aversion [20]. Research further demonstrates that chronic pain patients exhibit abnormally heightened connectivity between the amygdala and the central executive network, particularly in individuals with the highest levels of pain catastrophizing [21]. Therefore, based on the aforementioned mechanisms, for patients with pain catastrophizing, if we aim to alleviate both pain and improve catastrophic thinking, the combination of PRF and SGB appears particularly necessary. This combined therapeutic approach may disrupt the “pain‐sympathetic hyperactivity‐cognitive distortion” vicious cycle in patients with acute herpes zoster–associated neuralgia, thereby more effectively alleviating pain and psychological distress in this population.

Our results indicated that although the advantages of the combined therapy in VAS and PSQI scores gradually diminished after 3 months and showed no statistical difference from the PRF monotherapy group at 6 months, the combined treatment group consistently exhibited significantly lower PCS scores at all postoperative follow‐up time points compared with the PRF monotherapy group. This temporal pattern suggests that the clinical advantage of PRF + SGB is more prominent in the early post‐treatment period, particularly for pain intensity and sleep quality, rather than representing sustained long‐term analgesic advantage at 6 months. This discrepancy likely reflects the shorter duration of SGB’s analgesic effects in pain management [22], which may synergistically augment PRF’s efficacy during the early phase. PRF promotes neural repair and neuroplastic changes, requiring a longer period to reach peak effectiveness [23, 24], implying that long‐term analgesia may rely more heavily on PRF therapy. Consistent with this interpretation, the multivariable regression analyses showed that the treatment group was not independently associated with 6‐month VAS or PSQI after adjustment for age, disease duration, baseline VAS, baseline PCS, and baseline PSQI. In contrast, the treatment group remained significantly associated with 6‐month PCS, suggesting that the combined intervention was independently related to better long‐term improvement in pain catastrophizing rather than sustained advantage in pain intensity or sleep quality. Notably, the sustained improvement in pain catastrophizing in the combined group may reflect the central affective modulation mediated by SGB. This suggests that early implementation of SGB during the acute phase could rapidly disrupt the “pain‐sympathetic hyperactivity‐psychological stress” cycle, particularly for patients with significant psychological comorbidities. Early SGB not only alleviates acute stress responses but may also induce lasting cognitive improvements through limbic system neuroplasticity remodeling, potentially reducing long‐term psychological morbidity when administered during critical windows of neural adaptability [11, 13].

Regarding safety, neither group experienced severe complications, attributable to PRF’s nonablative nature and precision‐guided techniques. High‐resolution ultrasound‐guided supraorbital PRF visualization minimizes supraorbital artery injury risks, while real‐time ultrasound‐guided SGB significantly reduces recurrent laryngeal nerve block complications [25]. These technological advancements ensure the combined therapy’s safety profile.

As a retrospective investigation, this study has inherent limitations: First, treatment allocation was not randomized, and the choice of PRF alone or PRF + SGB may have been influenced by clinical characteristics, symptom severity, physician judgment, or patient preference. Therefore, selection bias cannot be excluded. Although multivariable regression was performed to adjust for potential confounders, including age, disease duration, and baseline VAS, PCS, and PSQI scores, residual confounding from unmeasured factors may still exist. The moderate sample size and restricted follow‐up duration necessitate larger‐scale longitudinal validation. Second, whether pain catastrophizing improvements directly correlate with specific neural circuit modifications requires confirmation through neuroimaging studies. Furthermore, the exclusion of cognitive behavioral therapy as a comparative psychological intervention suggests future research should explore multimodal therapeutic synergies. Prospective randomized controlled studies are needed to confirm whether the addition of SGB produces causal benefits and to determine whether its effects are primarily short‐term, long‐term, or outcome‐specific.

In summary, our findings suggest that combining PRF with SGB was associated with better early pain and sleep outcomes, and a more sustained reduction in pain catastrophizing, compared with PRF alone. However, the diminishing group differences at 6 months indicate that the combined approach primarily offers an early clinical advantage, while the persistent association with reduced catastrophizing may point to a distinct role of SGB in modulating cognitive–emotional processing. These observations, derived from a retrospective design, remain hypothesis‐generating and require confirmation through prospective randomized trials incorporating neuroimaging and multimodal therapy comparisons.

## 5. Conclusion

PRF targeting the supraorbital nerve combined with SGB was associated with greater short‐term pain relief, sleep improvement, and reduction in pain catastrophizing in acute HZTN patients with catastrophizing compared with PRF alone. However, the advantages in VAS and PSQI diminished over time, with no significant between‐group differences observed at 6 months, suggesting that the combined therapy mainly provides early clinical benefit rather than sustained long‐term analgesic or sleep‐related advantage. In contrast, the sustained reduction in catastrophizing scores and the adjusted regression results suggest a persistent association between PRF + SGB and improved pain catastrophizing, supporting the potential role of SGB in modulating emotional–cognitive dysfunction. This safe, ultrasound‐guided approach may be a clinically feasible adjunctive strategy for patients with prominent pain catastrophizing, but the retrospective, nonrandomized design limits causal inference. Larger prospective randomized trials and neuroimaging studies are needed to validate long‐term efficacy, clarify underlying mechanisms, and optimize treatment strategies.

## Funding

The study was funded by the Affiliated Hospital of Southwest Medical University (2024LCYXZX66).

## Conflicts of Interest

The authors declare no conflicts of interest.

## Data Availability

The data that support the findings of this study are available from the corresponding author upon reasonable request.
